# Awake Videolaryngoscopy for Intubation in Patients With Laryngeal Cancer: A Case Series

**DOI:** 10.7759/cureus.62993

**Published:** 2024-06-23

**Authors:** Stefano Barbaro, Pierdomenico Carone, Laura Lanotte, Ester Scapini, Michele Debitonto

**Affiliations:** 1 Anesthesia, Resuscitation and Pain Therapy, Ospedale Monsignor Dimiccoli, Barletta, ITA; 2 Medical Oncology, Ospedale Monsignor Dimiccoli, Barletta, ITA

**Keywords:** orotracheal intubation, total laryngectomy, advanced airway management, difficult airway management, awake intubation

## Abstract

Total laryngectomy is the gold standard surgical approach for laryngeal cancer and is generally conducted under general anesthesia. Orotracheal intubation remains a very delicate step in the general anesthesia process. In otolaryngology (ENT) surgery, it remains considered the preferred method of anesthesia for many surgical procedures. A significant challenge in oncological ENT surgery is the difficulty associated with orotracheal intubation, due to a number of reasons that can lead to failure of orotracheal intubation. To mitigate this risk, experts recommend proceeding with orotracheal intubation with the patient awake and breathing spontaneously. In this case series, we report four patients with supraglottic tumors of the larynx who underwent total laryngectomy surgery under general anesthesia, during which they underwent orotracheal intubation while awake and spontaneous breathing, under no sedative drugs of any kind, in order to avoid complications of orotracheal intubation failure and respiratory apnea due to bleeding tumor masses that engaged the supraglottic space.

## Introduction

To date, laryngeal cancer requires multidisciplinary treatment involving specialists such as otolaryngologists, oncologists, and radiation therapists. As far as surgical treatment is concerned, the gold standard is a total laryngectomy. From an anesthesiological point of view, the presence of a laryngeal tumor can lead to potential difficulties in tracheal intubation [1]. In patients with laryngeal cancer, there is a high risk of losing airway control during the induction of general anesthesia before tracheal intubation, either due to loss of hypopharyngeal muscle tone or dynamic/complete airway obstruction [2]. In the presence of such difficult airways, awake tracheal intubation (ATI) is considered the technique of choice [3]. Among the most commonly used methods for awake orotracheal intubation are orotracheal intubation with a fiberscope, either orally or nasally, and orotracheal intubation with a videolaryngoscope [4]. Awake orotracheal intubation increases patient safety. Keeping the patient awake and breathing spontaneously in this phase of the induction of general anesthesia helps avoid periprocedural complications, enables evaluation of other secondary options in case of intubation failure, and prevents excessive stress on the anesthesia team in case of complications [5]. The choice of the device should be based on the anesthetist's comfort and proficiency, with the best option being the instrument with which they are most familiar. The avoidance of sedative drugs such as benzodiazepines, hypnotics, or opioids is an obvious advantage of this anesthetic strategy, as it helps prevent respiratory apnea, airway obstruction, and loss of consciousness, which could result in loss of airway control [6]. This anesthetic procedure can also aid in various surgical interventions where general anesthesia is indicated, especially in the presence of signs predicting difficulty in orotracheal intubation [7].

## Case presentation

We report on four American Society of Anesthesiology Physical Status 3 patients with indications for laryngectomy.

For Case 1, the patient was an 82-year-old male smoker (20 cigarettes per day for 60 years) with hypertensive heart disease and chronic obstructive pulmonary disease (COPD), diagnosed with a supraglottic laryngeal tumor by a previous biopsy and not amenable to conservative treatment.

For Case 2, the patient was a 70-year-old male heavy smoker (40 cigarettes per day for 40 years) with COPD and a history of percutaneous transluminal coronary angioplasty (PTCA), diagnosed with a supraglottic laryngeal tumor by a prior biopsy and not amenable to conservative treatment.

For Case 3, the patient was a 79-year-old male heavy smoker (25 cigarettes per day for 40 years) with COPD and a history of two PTCAs in the last decade, diagnosed with supraglottic laryngeal tumors by prior biopsies, not amenable to conservative treatment.

For Case 4, the patient was an 83-year-old male heavy smoker (20 cigarettes per day for 60 years) with COPD, hypertensive heart disease, and a history of an aorto-coronary bypass 15 years earlier, diagnosed with a supraglottic laryngeal tumor by a previous biopsy, not amenable to conservative treatment.

**Figure 1:**
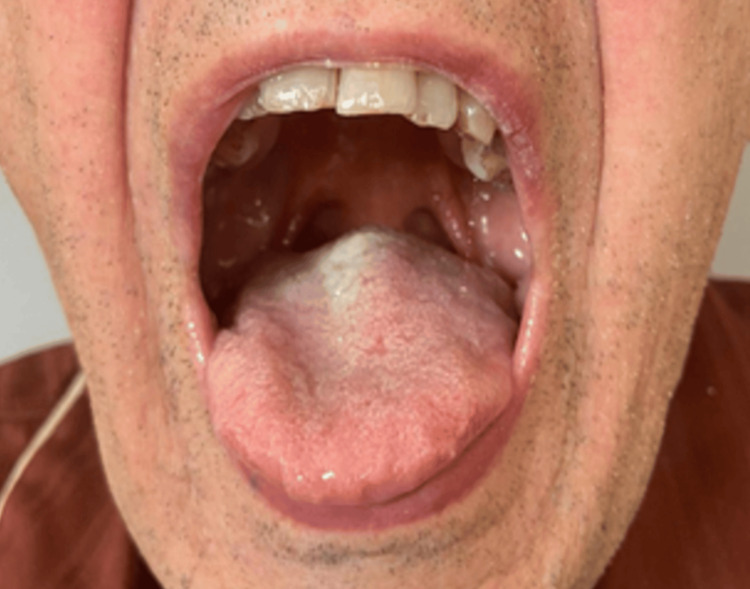
Case 1 evaluation of the Mallampati score: visualization of only the base of the uvula (grade 3)

The symptom common to all patients was hoarseness. During pre-admission, the patients underwent routine blood chemistry tests, cardiology workups, and chest and neck CT scans. The patients were examined by an anesthesiologist the day before the operation, with particular attention paid to the evaluation of the airways. The Simplified Airway Risk Index score was evaluated: mouth opening, thyromental distance, Mallampati score, movement of the neck, the ability to create an underbite, body weight, and previous intubation history. Additionally, a fiberoscopy was performed by the otolaryngologist to evaluate the respiratory space. In Figure 1, there is the Mallampati Case 1 score evaluation.

**Figure 2:**
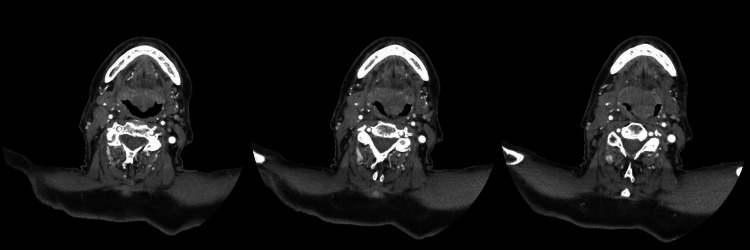
Case 1 CT scan of the neck showed swelling in the right median-paramedian site with homogeneous density, involving the lingual surface of the epiglottis and the root of the tongue. The glottic plane is asymmetrical, with thickening of the posterior and left lateral walls of the larynx in the supraglottic position CT: computed tomography

All four patients had an interdental distance greater than 35 millimeters; thus, we chose to proceed with the use of the videolaryngoscope instead of the fiberscope. These patients, all considered candidates for general anesthesia, presented a reduced respiratory space on fibroscopy, with a tumor mass partially obstructing this space as well as fragile tissue. Notably, in Case 1, an axial CT scan of the neck revealed the presence of supraglottic cancer at three different levels (Figure 2).

Awake orotracheal intubation without sedation has demonstrated a very high safety profile. In the operation room, heart rate, non-invasive blood pressure, continuous electrocardiogram, and oxygen saturation were monitored. All patients were pre-oxygenated with oxygen in nasal goggles at 15 liters per minute. Topicalization of the airway was done with 10% lidocaine sprayed during inspiration over the oropharynx, tonsillar pillars, and base of the tongue (four puffs on each side). Four milliliters (ml) of 2% lidocaine with adrenaline 0.1 mg were administered by aerosol. One gram of tranexamic acid intravenously in 100 ml of a 0.9% NaCl solution was administered intravenously to all patients. The lidocaine dose was kept below 9 mg per kg. No sedative or hypnotic drugs were administered during the awake intubation procedure to avoid drug-related apnea complications. The videolaryngoscope was inserted, and the endotracheal tube was gently positioned in a single shot. The tube was connected to the ventilator, and capnography was monitored. Standard anesthetic drugs were used for the induction and maintenance of general anesthesia. In all four cases, the operations proceeded without intraoperative or postoperative complications. At the end of all surgeries, the patients were awakened from general anesthesia, and after monitoring for about 30 minutes, they were transferred to the inpatient ward. Their postoperative course was uneventful, with standard days of hospitalization.

## Discussion

Adopting a strategy for probable difficult orotracheal intubation is of paramount importance. ATI involves the placement of the endotracheal tube with the patient alert and conscious, breathing spontaneously, possibly collaborating, with the use of a fiberscope or a videolaryngoscope [4]. The prediction of difficult airway management is not always accurate, but there are peculiarities in patients who could benefit from ATI. Indications include head and neck pathology (including malignancies, previous surgery, or radiotherapy), reduced buccal opening, patients with limited neck extension, patients suffering from obstructive sleep apnea syndrome, and severe obesity [5]. There are few contraindications to ATI, such as allergy to local anesthetics, airway bleeding, and uncooperative patients, while the absolute contraindication is patient refusal [4]. ATI in spontaneous breathing has a failure rate of 1-2% [5]. ATI is associated with a significant amount of psychological stress for the anesthesiologist and his team due to the difficulty in airway management [6]. These stressors can be associated with underperformance, thereby increasing the risk of failure [7]. Obviously, monitoring patients' vitals during anesthetic care mitigates risks and can alert caregivers to any impending complications. The anesthetist needs to be aware of the risk of excessive sedation, which can lead to airway obstruction and respiratory apnea [8]. Additionally, disorders of the heart rhythm and blood pressure may occur due to local anesthetic intoxication after topicalization [9]. The likelihood of success depends on the effective topical application of local anesthetics to the airways [10]. Lidocaine has theoretical safety advantages over other local anesthetic drugs due to its cardiovascular effect and relatively low risk of systemic toxicity, making it the most commonly used local anesthetic for ATI. The dose of topical lidocaine should not exceed 9 mg per kg of body weight [11]. ATI can be performed with or without sedation [12]. Various sedative drugs may produce a range of effects that may be considered desirable (e.g., increased patient compliance) or undesirable (e.g., excessive sedation). The risk of excessive sedation and its consequences, including respiratory depression, airway obstruction, hypoxia, aspiration, and cardiovascular instability, can adversely affect patient outcomes. Thus, it is advised to limit the use of these sedative drugs [12]. Once the correct positioning of the endotracheal tube has been visualized and the correct capnography has been obtained, general anesthesia can be initiated. Patients considered candidates for ATI are at higher risk of adverse events associated with multiple intubation attempts, airway trauma, airway obstruction, bleeding, and heart failure [13]. In cases of three failed attempts, the administration of 100% oxygen should be put first, and the administration of potentially antagonistic drugs should be stopped. Ideally, in the event of a failed ATI, the preferred action is to postpone the procedure [12]. In cases of emergency, it is advised to proceed with the orotracheal intubation protocol while considering the possibility of proceeding with an emergency cricothyroidotomy or tracheostomy [4].

## Conclusions

During the procedure, a high degree of patient cooperation was noted in all four cases. There were no reported complications. The presence of an expert anesthetist is recommended for the possibility of periprocedural complications. During ATI, the natural airway is better maintained and the muscle tone is present which keeps the structures separate, making them better identifiable, and the larynx is in a more favorable position. Awake videolaryngoscopy intubation is an excellent strategy to adopt in patients with laryngeal cancer, particularly when expecting potentially complicated intubation.
